# Mortality among Hospitalized Dengue Patients with Comorbidities in Mexico, Brazil, and Colombia

**DOI:** 10.4269/ajtmh.20-1163

**Published:** 2021-05-10

**Authors:** Alejandro E. Macias, Guilherme L. Werneck, Raúl Castro, Cesar Mascareñas, Laurent Coudeville, David Morley, Vincent Recamier, Mariana Guergova-Kuras, Adrien Etcheto, Esteban Puentes-Rosas, Nicolas Baurin, Myew-Ling Toh

**Affiliations:** 1 Área De Microbiología, Departamento De Medicina y Nutrición, Universidad de Guanajuato, Guanajuato, Mexico;; 2 Instituto de Estudos em Saúde Coletiva, Universidade Federal do Rio de Janeiro, Rio de Janeiro, Brazil;; 3 Los Andes University, Bogota, Colombia;; 4 Sanofi Pasteur, Lyon, France;; 5 Ariana Pharmaceuticals, Paris, France;; 6 Sanofi Pasteur, Mexico City, Mexico

## Abstract

Dengue patients with comorbidities may be at higher risk of death. In this cross-sectional study, healthcare databases from Mexico (2008–2014), Brazil (2008–2015), and Colombia (2009–2017) were used to identify hospitalized dengue cases and their comorbidities. Case fatality rates (CFRs), relative risk, and odds ratios (OR) for in-hospital mortality were determined. Overall, 678,836 hospitalized dengue cases were identified: 68,194 from Mexico, 532,821 from Brazil, and 77,821 from Colombia. Of these, 35%, 5%, and 18% were severe dengue, respectively. Severe dengue and age ≥ 46 years were associated with increased risk of in-hospital mortality. Comorbidities were identified in 8%, 1%, and 4% of cases in Mexico, Brazil, and Colombia, respectively. Comorbidities increased hospitalized dengue CFRs 3- to 17-fold; CFRs were higher with comorbidities regardless of dengue severity or age. The odds of in-hospital mortality were significantly higher in those with pulmonary disorders (11.6 [95% CI 7.4–18.2], 12.7 [95% CI 9.3–17.5], and 8.0 [95% CI 4.9–13.1] in Mexico, Brazil, and Colombia, respectively), ischemic heart disease (23.0 [95% CI 6.6–79.6], 5.9 [95% CI 1.4–24.6], and 7.0 [95% CI 1.9–25.5]), and renal disease/failure (8.3 [95% CI 4.8–14.2], 8.0 [95% CI 4.5–14.4], and 9.3 [95% CI 3.1–28.0]) across the three countries; the odds of in-hospital mortality from dengue with comorbidities was at least equivalent or higher than severe dengue alone (4.5 [95% CI 3.4–6.1], 9.6 [95% CI 8.6–10.6], and 9.0 [95% CI 6.8–12.0). In conclusion, the risk of death because of dengue increases with comorbidities independently of age and/or disease severity.

## INTRODUCTION

Dengue incidence has increased 30-fold in the last 50 years, with geographic expansion to new countries and, more recently, from urban to rural settings.^1^ The disease is currently endemic in more than 100 countries, with the Americas, Southeast Asia, and the Western Pacific the most affected regions.^2^ The Americas had 14% (13 million infections) of apparent dengue infections worldwide in 2010, over half of which occurred in Brazil and Mexico.^3^ In 2017, there were 89,893 notified dengue cases in Mexico, 252,054 in Brazil, and 26,279 in Colombia.^4^ However, the true magnitude of the dengue burden is likely underestimated.^5^ Early detection and access to medical care can reduce fatality rates to less than 1%.^2^ Between 2014 and 2017, the annual dengue case fatality rate (CFR) ranged from 0.02% to 0.04% in Mexico, 0.04% to 0.07% in Brazil, and 0.06% to 0.16% in Colombia.^4^

There is no specific treatment for dengue. In dengue endemic regions, preventative measures include vector control, avoidance of getting bitten, and vaccination. The recombinant, live, attenuated, tetravalent dengue vaccine (Dengvaxia^®^; CYD-TDV)^6^ is indicated for the prevention of dengue disease in individuals confirmed to be dengue-seropositive aged 9–16 years or 9–45 years depending on specific country/regional approval.^7,8^ Individuals who are dengue-seronegative should not be vaccinated, as they are at increased risk of severe dengue following vaccination. Currently, the dengue vaccine is registered in 19 countries in Asia and Latin America, as well as in eligible parts of the European Union and the United States.^9^

Underlying chronic disorders may have the potential to contribute to the severity of physiological responses to dengue infection or *vice versa* (i.e., the physiological responses to dengue infection may exacerbate some pre-existing comorbidities), resulting in a worse outcome. A number of small, retrospective, case-control, and case-review studies have identified some comorbidities as possible risk factors that might influence development of severe dengue and dengue-related mortality.^10–15^ However, there have been few large-scale studies assessing the impact of comorbidities on the CFR from dengue. The aims of this study were to examine dengue-related hospitalization and CFRs in Mexico, Brazil, and Colombia using health system databases, and to assess the impact of comorbidities on in-hospital dengue mortality. A greater understanding of the role of underlying comorbidities in the development of severe outcomes would help better target dengue vaccination strategies as well as clinical monitoring to ensure prompt, aggressive supportive therapy for those at high-risk, and thus lead to a reduction of dengue-related mortality.

## METHODS

### Data sources.

Anonymized data from three health system databases were used in this study. The Mexican Subsistema Automatizado de Egresos Hospitalarios (SAEH) is the main hospital discharge database for all Ministry of Health hospitals in Mexico, representing 38.3% of total services provided in the country.^16^ During the study period 2008–2014, the SAEH database included data on 19.2 million hospital admission records from 817 hospitals.^17^ We previously used the same dataset in another analysis assessing the burden of dengue on hospital services in Mexico.^18^ The Brazilian Hospital Information System of the Unified Health System (SIH/SUS) covers 70–80% of hospital admissions in Brazil. During the study period 2008–2015 the SIH/SUS database included data on 92 million hospital admission records from 5,983 hospitals.^19^ The Colombian Registro Individual de Prestaciones de Salud (RIPS) database, maintained by the Colombian Ministry of Health, contains information regarding hospitalizations, services, and supplies provided, as well as medicine and outpatient care. During the study period 2009–2017, the RIPS database included data on 13.5 million hospital admission records from 11,208 hospitals (approximately 70–75% of the services provided).

Dengue is a notifiable disease in the three countries. Primary and secondary diagnosis, based on the International Classification of Diseases, 10th Revision (ICD-10) codes^20^ were used across all three countries to identify dengue cases and comorbidities from the databases. Dengue cases were classified as either non-severe (ICD-10 code = A90; classical dengue) or severe dengue (ICD-10 code = A91; dengue hemorrhagic fever); the dengue diagnosis code position (primary or secondary) was not taken into consideration in this analysis. The number of available fields for reporting of secondary diagnosis codes varied by country, and appeared as follows over the duration of the study in Brazil and Mexico: Brazil (2008–2014: 1 field; 2015: 9 fields); Mexico (2008–2009: 6 fields; 2010–2014: unlimited fields); Colombia (3 fields). For consistency within each country analysis, only the first secondary code was considered for all years in Brazil; there were up to six secondary codes considered for Mexico, and up to three secondary codes for Colombia. Comorbidities were identified from a preliminary analysis of ICD-10 codes (the first three characters) associated with in-hospital mortality in patients with dengue. All identified ICD-10 codes for comorbidities were then grouped into larger categories: diabetes, HIV, heart failure, hypertension, ischemic heart disease, dyslipidemia, obesity, pulmonary disorders, renal disease or failure, stroke, urinary disorders, and infectious diseases (excluding dengue) (Supplemental Table S1). To exclude potential bias in the analysis, codes considered as symptoms of dengue or severe dengue and its complications were excluded, such as fever, headache, and dehydration.

### Outcome measures.

We recorded the number of cases of hospitalized dengue, non-severe and severe, and the proportion of cases with a specified comorbidity, in each of the Mexican, Brazilian, and Colombian databases. CFRs were calculated as the proportion of recorded cases of dengue that were fatal during the study period (only in-hospital mortality during the same hospitalization was considered), and they were calculated separately for non-severe and severe dengue cases with and without comorbidities. Relative risk (RR) was calculated as the ratio of the CFRs in hospitalized dengue cases with comorbidities to those without comorbidities. Odds ratios (ORs) were calculated to determine the impact of comorbidities, dengue severity, age on admission, and year of admission on in-hospital mortality and intensive care unit (ICU) admission. ORs were derived from multivariate regression analysis, whereas RRs were based on the univariate analysis; of note, ORs are like RRs when the event is rare.

### Statistical analyses.

Patients were stratified according to age on hospital admission: 0–8 years, 9–45 years, 46–60 years, or ≥ 61 years. The RR and associated 95% confidence intervals (CIs) were calculated according to standard formulae,^21^ and *P* values were calculated using Fisher’s exact tests. To ensure reliability/robustness of estimated CFRs and RRs, comorbidities by age group were only reported for age groups with at least five cases and one death, and a *P* value < 0.05 (Fisher’s exact test).

The effect of risk factors on the binary outcome measures of in-hospital mortality and ICU admission were examined in random-effects multivariate logistic regression models, including a random intercept for hospitals. Risk factors included in the multivariate logistic regression models were comorbidities identified in the univariate analysis in at least two age groups in at least two databases. Infectious diseases were excluded to prevent potential confounding effects because of the presence of differential diagnoses for dengue/other infections in this category. Dengue severity, age, and year of admission were also included in the models. The age stratum of the patient on admission (the reference category was age 9–45 years, which corresponds to the indicated age for dengue vaccination in Mexico and Brazil [the vaccine is not yet registered in Colombia]) and the year of admission in the database (2008 in Mexico and Brazil, and 2009 in Colombia as reference category) were also included in models to adjust for the potential confounding effects of patient age and admission year. The coefficients derived from these logistic regressions were exponentiated to obtain adjusted ORs and associated 95% CIs and *P* values.

Analyses were performed using the KNIME^22^ analytic platform (Knime: 3.5.2 integrated with KEM^®^,^23^ Ariana Pharmaceuticals) data mining tools, MySQL database and R statistical software, the *glm* function of the stats base package of the R statistical software (R: 3.4.3 “Kite-Eating Tree”), and the *melogit* command for multilevel mixed-effects logistic regression in Stata 15.1^®^.

## RESULTS

### Cases of hospitalized dengue.

Overall, 678,836 hospitalized dengue cases were identified in the three databases assessed across the three countries. There were 68,194 hospitalized dengue cases identified from the Mexican database during 2008–2014, of which 44,357 (65%) were reported as non-severe dengue and 23,837 (35%) as severe dengue; and there were 267 in-hospital deaths among these cases (Supplemental Table S2). In the Brazilian database, 532,821 hospitalized dengue cases were identified during 2008–2015, of which 505,697 (95%) were reported as non-severe dengue and 27,124 (5%) as severe, and there were 2698 in-hospital deaths among these cases (Supplemental Table S3). From the Colombian database, 77,821 hospitalized dengue cases were identified during 2009–2017, of which 63,579 (82%) were reported as non-severe dengue and 14,242 (18%) as severe, and there were 260 in-hospital deaths among these cases (Supplemental Table S4).

### Prevalence of comorbidities.

**Figure 1.**
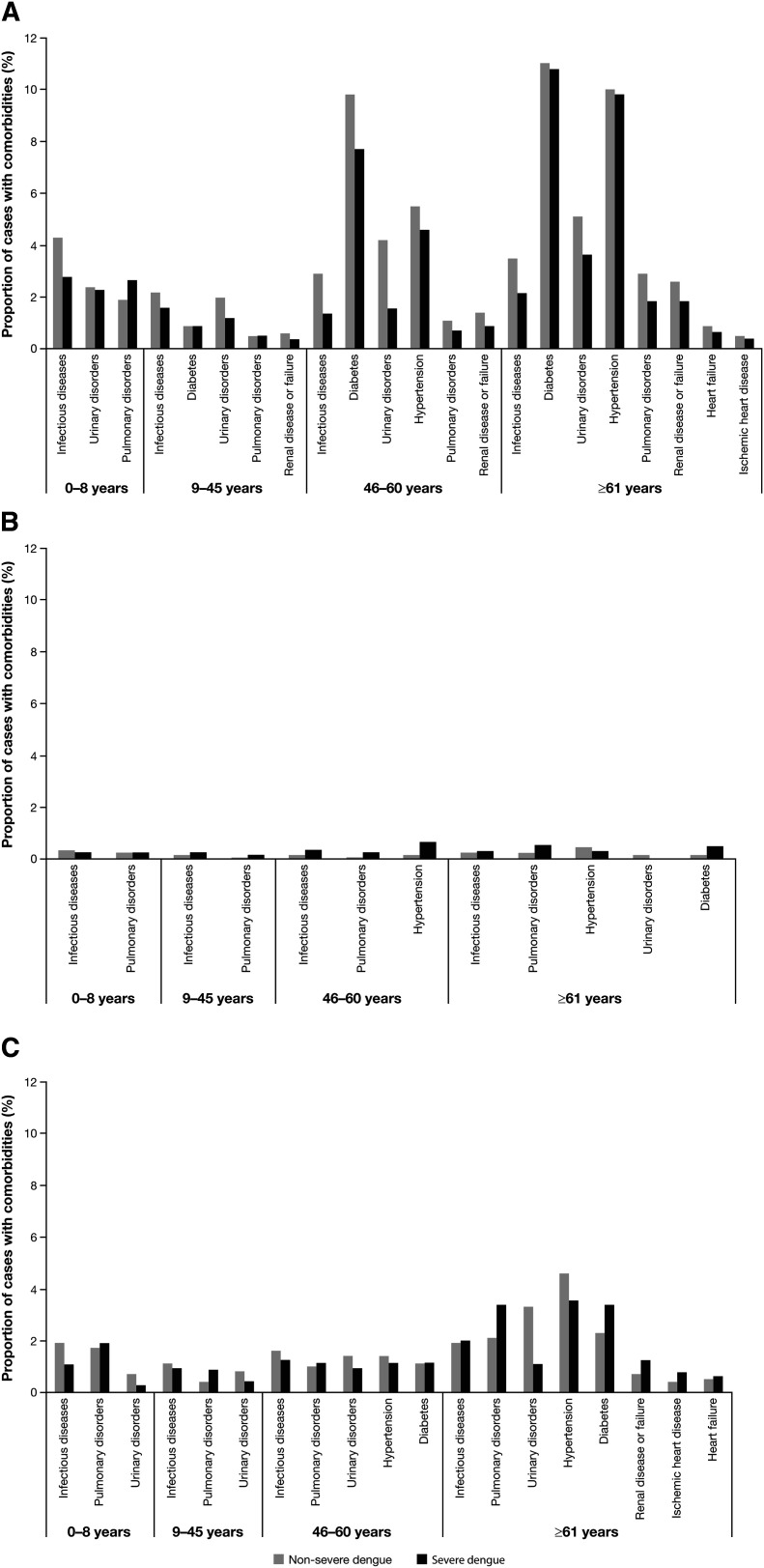
Summary of hospitalized dengue cases with comorbidities in **A**) Mexico, **B**) Brazil, and **C**) Colombia. Only comorbidities with frequencies ≥ 0.5% in Mexico and Colombia, or ≥ 0.2% in Brazil are shown.

Of the hospitalized dengue cases in Mexico, there was an additional diagnosis of at least one of the specified comorbidities in 4,047 (9%) of the non-severe dengue cases and 1,672 (7%) of the severe dengue cases (Supplemental Table S2). In Brazil, comorbidities were seen in 3,721 (0.7%) of the non-severe dengue cases and 283 (1%) of the severe cases (Supplemental Table S3); and in Colombia, comorbidities were seen in 2,505 (4%) of the non-severe cases and 474 (3%) of the severe cases (Supplemental Table S4). In general, the prevalence of comorbidities was lowest in the 9- to 45-year age group and increased with age (Figure 1). Comorbidities with the highest prevalence in all three countries were other infectious diseases (Supplemental Table S5 summarizes the top [accounting for 95% of codes] ICD-10 codes in the infectious disease A00–A99 comorbidity category reported in this study), diabetes, urinary disorders, pulmonary disorders, and hypertension (Supplemental Tables S2, S3, and S4). When the type of comorbidity was compared between hospitalized dengue cases and other hospitalized non-dengue cases, there was a much higher prevalence of other infectious diseases, pulmonary disorders, and urinary disorders among dengue cases (Supplemental Figure S1).

### Case fatality rates.

**Figure 2.**
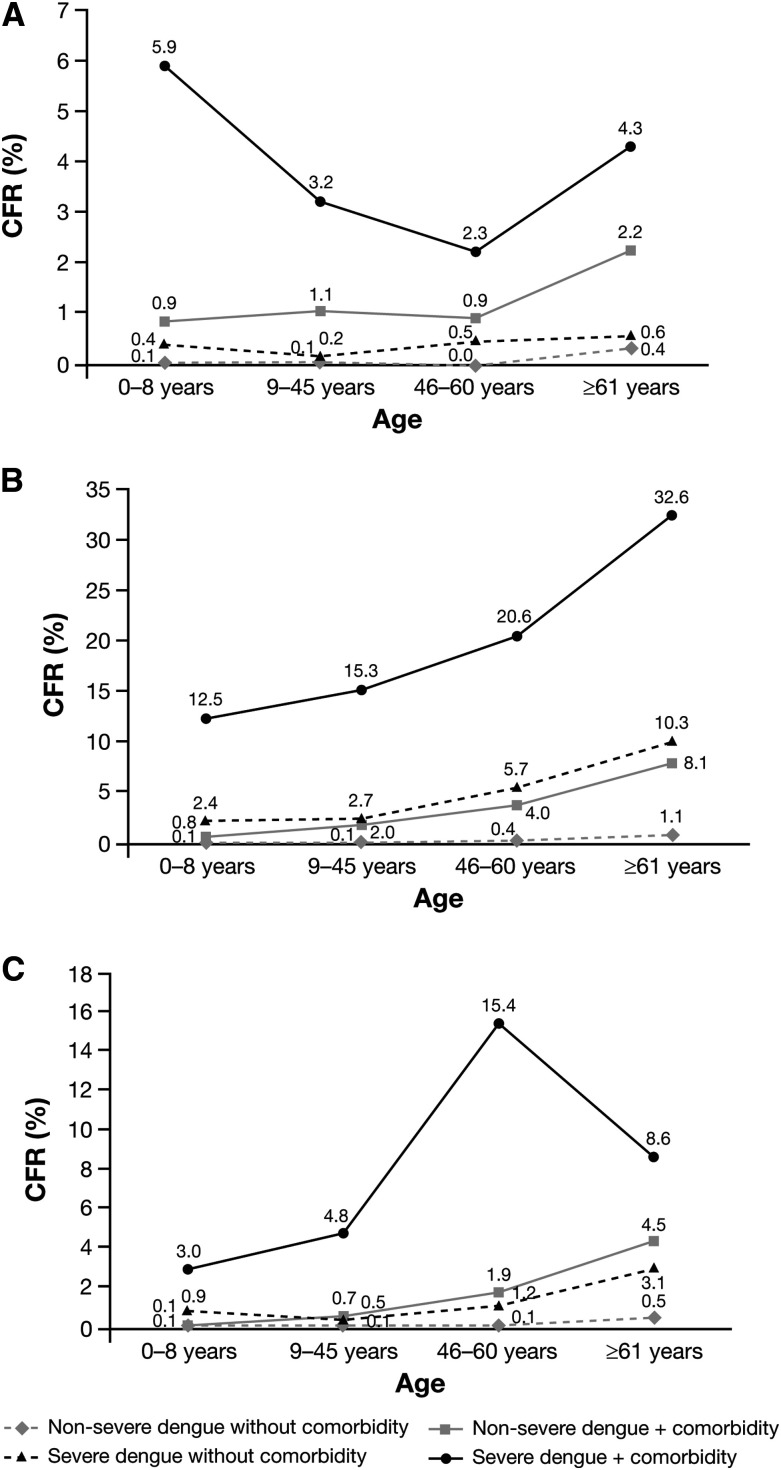
Case fatality rates for hospitalized dengue patients with nonsevere dengue and severe dengue diagnoses, with and without comorbidities, in Mexico (**A**), Brazil (**B**), and Colombia (**C**), stratified by age group. CFR = case fatality rate (proportion of reported cases of dengue that were fatal during the study period).

**Figure 3.**
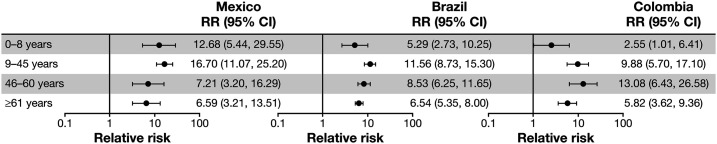
Relative risk of in-hospital mortality for all hospitalized dengue cases with comorbidities relative to cases with no comorbidities, stratified by age group. Shown are relative risks for all dengue cases, nonsevere, and severe (proportion of reported cases of dengue that were fatal during the study period for cases with dengue with comorbidity vs cases with dengue alone).

The CFRs for hospitalized dengue were higher in the presence of common comorbidities in Mexico, Brazil, and Colombia, regardless of dengue severity or age (Figure 2). However, the highest CFRs were seen in individuals with both severe dengue and comorbidities in the different age groups, reaching 5.9% for the 0- to 8-year age group in Mexico, 32.6% for the ≥ 60-year age group in Brazil, and 15.4% for the 46- to 60-year group in Colombia. In comparison, CFRs for severe dengue without comorbidities across the age groups were 0.4–0.6%, 2.4–10.3%, and 0.5–3.1% in Mexico, Brazil, and Colombia, respectively. Comorbidity with renal disease or failure, pulmonary disorders, and infectious diseases increased hospitalized dengue CFR at any age in Mexico, which was also the case with renal disease or failure, and infectious diseases in Brazil (Supplemental Table S6). The RR of death among hospitalized dengue patients with comorbidities to those without comorbidities was higher across all ages, with CFRs 7–17 times higher in Mexico, 5–12 times higher in Brazil, and 3–13 times higher in Colombia (Figure 3).

### Impact of risk factors on outcomes.

**Figure 4.**
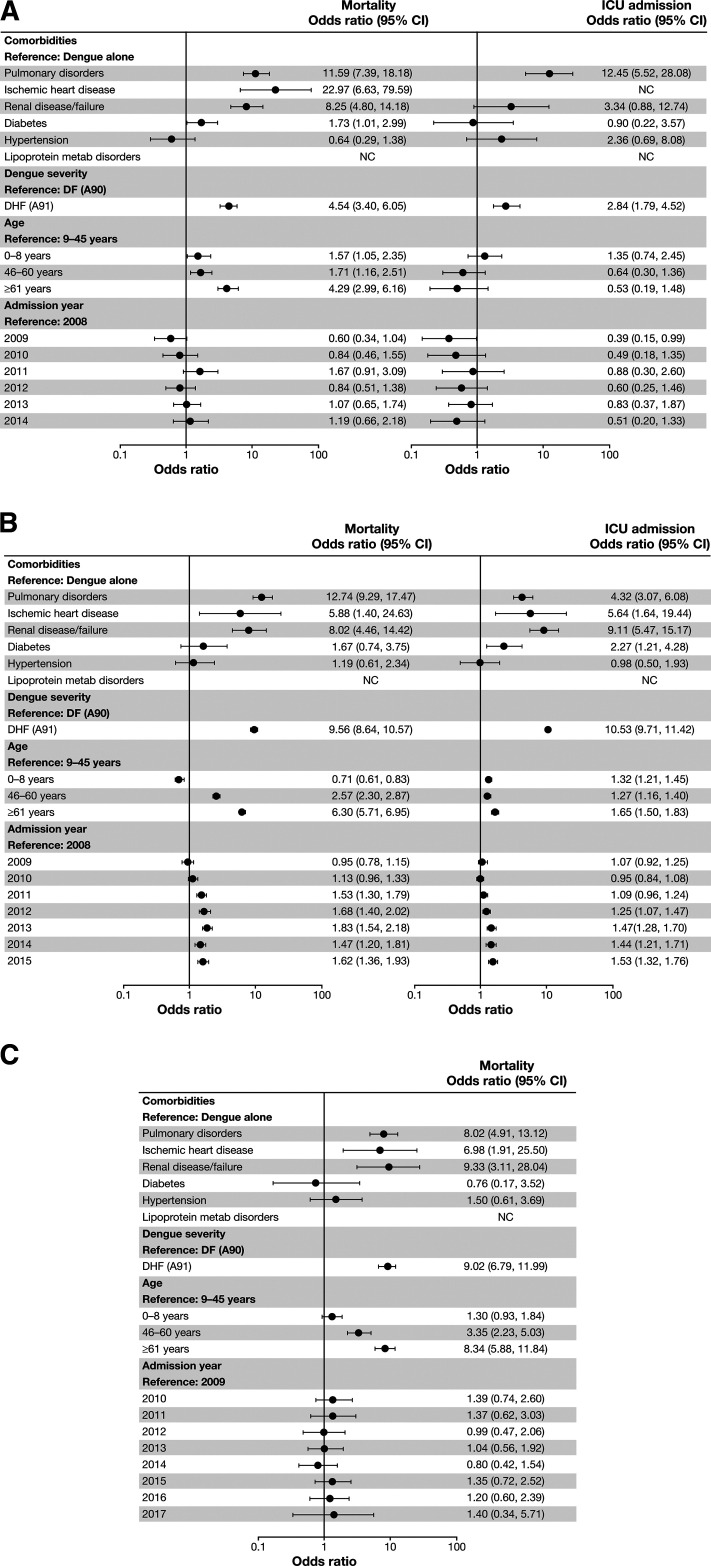
Adjusted odds ratios for mortality and ICU admission in Mexico (**A**), Brazil (**B**), and Colombia (**C**). NC = noncalculable. Risk factors included in the multivariate logistic regression models were comorbidities identified in the univariate analysis in at least two age groups in at least two databases. Infectious diseases were excluded to prevent potential confounding effects because of the presence of differential diagnoses for dengue/other infections in this category. Dengue severity, age, and year of admission were also included in the models.

The risk of in-hospital mortality was significantly higher among hospitalized dengue patients with pulmonary disorders, ischemic heart disease, and renal disease/failure comorbidities versus those without these comorbidities, and the risk was consistent across the three countries (Figure 4). Age ≥ 46 years at admission versus 9–45 years was also associated with higher risk for in-hospital mortality in all countries, with the greatest risk for the oldest group (≥ 61 years) (Figure 4). In Brazil, there was a higher risk for in-hospital mortality from 2011 to 2015 relative to 2008 (reference year).

Data on ICU admission were unavailable in Colombia. In general, ICU admission rates for dengue cases (all dengue cases, dengue only, or dengue with comorbidity) were 4.5- to 9.5-fold lower in Mexico than Brazil (Supplemental Table S2 and S3). Pulmonary disorders, ischemic heart disease, and renal disease/failure comorbidities were also significant risk factors for ICU admission, along with diabetes in Brazil (Figure 4). In Mexico, renal disease/failure and older age were not significantly associated with an elevated risk of ICU admission, which may be because of the small number of admissions over this period: 122 of 68,194 hospitalized dengue cases (Supplemental Table S2).

## DISCUSSION

Although the proportion of hospitalized dengue cases with associated comorbidities was relatively small in the three countries assessed, the impact on mortality was significant. The presence of comorbidities increased the CFRs of hospitalized dengue by 3- to 17-fold compared with cases with no comorbidities. Moreover, the CFRs for hospitalized dengue were higher in the presence of common comorbidities regardless of dengue severity or age. Crucially, our study showed that the risk of death in hospitalized dengue cases was consistently increased across the three countries in cases with comorbid pulmonary disorders, ischemic heart disease, and/or renal disease/failure. These comorbidities were also significant risk factors for ICU admission, along with diabetes in Brazil, but the data on ICU admissions were generally limited in Mexico or unavailable for Colombia. We confirm other studies demonstrating that renal failure or renal insufficiency^12,24^ and ischemic heart disease^25^ increase the risk of severe dengue and/or in-hospital mortality. In contrast, despite odds ratios above 1 in some cases, we were unable to confirm that diabetes,^11–13,25,26^ hypertension,^12,14^ secondary infectious diseases,^12^ asthma,^26^ and allergies^13,14^ significantly increased hospitalized dengue CFR. Given the increasing incidence of many of these comorbidities globally,^27^ and locally to Mexico, Brazil, and Colombia,^28^ the number of dengue cases with these comorbidities will likely increase in future years, leading to greater hospital resource use and cost, in addition to increasing in-hospital death rates. The global average cost (direct and indirect) per hospitalized dengue case was estimated (in 2013 United States dollars) to be $70.10, with costs varying by region depending on income, from $56 in low income regions to $1,146 in high income regions.^29^ The costs per fatal case are substantial, estimated at $84,730 for children and $75,820 for adults. Thus strategies that reduce hospitalized cases, and dengue-related mortality in particular, would likely lead to considerable cost savings.

We also showed that severe dengue was associated with an increased risk of in-hospital mortality and ICU admissions (where data was available) versus non-severe dengue in all three countries. Older age in individuals above 45 years was also a risk factor for in-hospital mortality, as well as ICU admissions in Mexico and Brazil. Older age has been previously shown to lead to higher fatality rates in dengue cases^12,30–32^ and increased length of hospital stay.^33^ In addition, the prevalence of comorbidities was lowest in the 9- to 45-year age group and increased with age, which predisposes the elderly to increased risk of dengue mortality. Of note, in Brazil, there was a higher risk of dengue mortality observed from 2011 to 2015 relative to 2008 (reference year) in our study. It is possible that the increased recirculation of dengue serotype 1 in 2010, after many years of relatively low circulation rates, may have increased the number of serious manifestations of the disease in the subsequent years.^34^ Differences in CFRs among the three countries may reflect differing practices in the management of dengue (or experience with the illness and resultant accuracy of ICD coding), as well as differing classification of severe dengue^35^ and temporal circulation of the dengue serotypes.

Differences among countries concerning treatment guidelines, decision to hospitalize a patient, and the resulting clinical profiles of hospitalized dengue cases could all affect in-hospital mortality. The results of our study give support to this possibility, because we observed substantial differences in age-specific CFRs across the three countries. However, the results of the multivariate analysis show that the direction and the strength of the association between mortality and comorbidities did not markedly vary among countries. This suggests that the potential variations in the clinical profile of hospitalized dengue cases did not substantially interfere in such associations, provided that the analysis was adjusted for age and dengue severity.

Several other studies have also implicated underlying comorbidities (in particular, pre-existing heart disease and diabetes) in severe outcomes of other arboviral diseases, including infection with West Nile, chikungunya, and tick-borne encephalitis viruses.^36–38^ In general, the etiological relationship between pre-existing comorbidities and disease severity remains to be fully elucidated. It is possible that the physiological responses to the viral infections may exacerbate some pre-existing comorbidities, but other comorbidities may contribute to the severity of the physiological responses to the infection.

There are several limitations to our study that need to be considered when making generalizations to other dengue endemic countries. The database used in Mexico captured mortality in public Ministry of Health hospitals only. In contrast, in the Brazilian database, hospitalizations were from public and private hospitals that provide services for the government, covering 70–80% of total hospital admissions in the country. However, the RIPS database included information from both private and public health provider institutions that are obliged to report the services and supplies provided to any patient (whether hospitalized or not) to the Colombian Ministry of Health. Thus, these findings may not be applicable across the broader Latin American population. In addition, the number of comorbidities reported for a hospital admission in Brazil was limited to one principal diagnosis and one secondary diagnosis for most of the study period assessed (see Methods), whereas multiple comorbidities were reported for Mexico and Colombia. The differences in the reporting of comorbidities (including reporting practices) may in part explain the 4- to 8-fold higher prevalence of comorbidities reported in the latter two countries compared with Brazil. Caution is encouraged in the interpretation of these data because of the relatively great uncertainty, as conveyed by the CIs, in the estimates provided.

The true in-hospital mortality because of dengue may also be underestimated because of variability in reporting requirements in the different countries and underdiagnosis owing to the nonspecific clinical presentation of the disease. In addition, the databases assessed were primarily for administrative/reimbursement purposes, and there was no independent validation or confirmation of the cases. Differences in CFRs among the three countries may reflect differing practices in the management of dengue (or experience with the illness and resultant accuracy of ICD coding), as well as differing classification of severe dengue,^35^ and temporal circulation of the dengue serotypes. For all countries, the analysis was based on a combination of clinical and/or laboratory (virologically confirmed) dengue diagnoses. Viral infectious diseases reported as a risk factor for in-hospital dengue mortality may be confounders in the analysis, and it is not clear if they represent co-infections (e.g., Zika, yellow fever, Chikungunya) or differential diagnoses for dengue/other infections. Ideally, the analysis should have examined bacterial, viral, parasitic, and tuberculosis infections separately, but this information was not available. There is potential for reporting bias as comorbidities are more likely to be documented, and more likely to be severe, in a hospital setting. The severity of comorbidities may bias the CFR but was not determined in this study. It is also possible that some of the reported comorbidities such as pulmonary disorders or renal disease/failure may have been a complication of dengue, but some of these complications may have been grouped as underlying chronic conditions. Nonetheless, our study highlights the importance of comorbidities in dengue deaths and the need for better protection measures against dengue infection for patients with comorbidities in the absence of specific antiviral treatments.

Effective allocation of resources to strategies such as vaccination and other general protection measures against mosquito bites such as vector control (social/environmental) or personal protection (use of protective clothing, insect repellent or nets), as well as surveillance for vector-borne infections will be important in preventing infections.^39^ Dengvaxia is currently the only licensed dengue vaccine to date, but its use is restricted to those with evidence of prior dengue infection(s) (i.e., dengue seropositive)^7,8^ so as to minimize the risk of severe dengue by avoiding vaccination of those without prior dengue infection (i.e., dengue seronegative).^40^ Thus, determining the recipient’s serostatus before administration of the vaccine remains a high priority.^41,42^ In addition, the vaccine has variable efficacy against the four dengue serotypes (lower for serotypes 1 and 2 than for serotypes 3 and 4),^43–45^ with an overall efficacy of 76% against symptomatic, virologically confirmed dengue up to 25 months after the first vaccination in those with evidence of prior dengue infection(s) aged ≥ 9 years.^40^ Nonetheless, the overall number of infections would likely be unaffected because only seropositives would be targeted for vaccination.^41,42,46^ A combination of sustained vector control and vaccination would be more effective in suppressing and maintaining the number of cases at very low levels than vaccination alone.^46^

In conclusion, our retrospective study demonstrates that the risk of death because of dengue in adult populations in Mexico, Brazil, and Colombia increases with comorbidities independently of age and/or disease severity. Worldwide, there is an increasing elderly population and high prevalence of comorbidities in dengue-endemic countries. These data support the need for prompt diagnosis and adequate care for the management of patients with comorbidities and dengue, as well as the use of preventive measures, such as dengue vaccination and vector control.^47^

## Supplemental table and figures

Supplemental materials
