# Supplementary material for: Mortality among Hospitalized Dengue Patients with Comorbidities in Mexico, Brazil, and Colombia
Source: Am J Trop Med Hyg. 2021 May 10;105(1):102–9. doi: 10.4269/ajtmh.20-1163 (PMC8274750; doi:10.4269/ajtmh.20-1163)
Supplement: Supplementary file 1 [file tpmd201163.SD1.pdf]

Supplemental materials

Table S1. ICD10 code definitions for comorbidity groups [International Statistical Classification of Diseases and Related Health Problems 10th Revision (ICD-10)-2014-WHO Version for 2014. http://apps.who.int/classifications/icd10/browse/2014/en]

Comorbidity Group	ICD10 Code	Description
**Diabetes**	E10	Insulin-dependent diabetes mellitus
	E11	Non-insulin-dependent diabetes mellitus
	E13	Other specified diabetes mellitus
	E14	Unspecified diabetes mellitus
**HIV**	B20	Human immunodeficiency virus [HIV] disease resulting in infectious and parasitic diseases
	B22	Human immunodeficiency virus [HIV] disease resulting in other specified diseases
	B23	Human immunodeficiency virus [HIV] disease resulting in other conditions
	B24	Unspecified human immunodeficiency virus [HIV] disease
**Heart failure**	I50	Heart failure
**Hypertension**	I10	Essential (primary) hypertension
	I11	Hypertensive heart disease
	I12	Hypertensive renal disease
	I15	Secondary hypertension
**Ischaemic heart disease**	I20	Angina pectoris
	I21	Acute myocardial infarction
	I24	Other acute ischaemic heart diseases
	I25	Chronic ischaemic heart disease
**Dyslipidemia**	E78	Disorders of lipoprotein metabolism and other lipidaemias
**Obesity**	E66	Obesity
**Pulmonary disorders**	J12	Viral pneumonia, not elsewhere classified
	J15	Bacterial pneumonia, not elsewhere classified
	J18	Pneumonia, organism unspecified
	J21	Acute bronchiolitis
	J40	Bronchitis, not specified as acute or chronic
	J44	Other chronic obstructive pulmonary disease
	J45	Asthma
	J90	Pleural effusion, not elsewhere classified
	J96	Respiratory failure, not elsewhere classified
**Renal disease or failure**	N10	Acute tubulo-interstitial nephritis
	N11	Chronic tubulo-interstitial nephritis
	N12	Tubulo-interstitial nephritis, not specified as acute or chronic
	N13	Obstructive and reflux uropathy
	N18	Chronic kidney disease
	N17	Acute renal failure
	N19	Unspecified kidney failure

Comorbidity Group	ICD10 Code	Description
**Stroke**	I64	Stroke, not specified as haemorrhage or infarction
**Urinary disorders**	N39	Other disorders of urinary system
**Infectious disease**[#]	A00–A99	Infectious diseases (excluding dengue A90/91)

[#]See Table S5 for the breakdown of the most frequent infectious disease codes.

Table S2. Summary of dengue cases for Mexico (SAEH 2008–2014)

	Cases	Severe Dengue	ICU Admission	In-hospital deaths	All dengue cases (deaths)				Severe dengue cases (A91)(deaths)			
					0–8	9–45	46–60	≥61	0–8	9–45	46–60	≥61
**All dengue cases**[1]	68,194	23,837	122	267	7311 (34)	50220 (142)	6887 (37)	3776 (54)	1972 (20)	17,951 (90)	2569 (23)	1345 (26)
**Dengue only**[2]	52,682	18,784	62	69	5426 (9)	40,084 (41)	4894 (9)	2278 (10)	1465 (6)	14,528 (24)	1928 (9)	863 (5)
**Dengue + any diagnosis**[3]	15,512	5053	60	198	1885 (25)	10,136 (101)	1993 (28)	1498 (44)	507 (14)	3423 (66)	641 (14)	482 (21)
**Dengue + comorbidity**[4]	5719	1672	19	107	618 (13)	2927 (50)	1206 (16)	968 (28)	152 (9)	865 (28)	355 (8)	300 (13)
Infectious diseases	1550	402	3	36	287 (7)	988 (23)	161 (2)	114 (4)	55 (5)	283 (11)	35 (1)	29 (1)
Diabetes	1504	505	3	20	5	466 (4)	621 (4)	412 (12)	1	161 (2)	198 (2)	145 (6)
Urinary disorders	1426	350	1	7	173 (1)	858 (2)	223 (1)	172 (3)	45 (1)	216	40 (1)	49 (3)
Hypertension	950	323	4	9	2 (1)	217 (1)	355 (1)	376 (6)		73 (1)	118 (1)	132 (2)
Pulmonary disorders	573	187	9	31	155 (4)	260 (14)	63 (5)	95 (8)	52 (3)	93 (8)	17 (2)	25 (2)
Renal disease or failure	440	119	3	23	23 (1)	246 (13)	84 (3)	87 (6)	2 (1)	68 (12)	24 (1)	25 (3)
HIV	113	41		6	2	95 (6)	16			36 (2)	5	
Obesity	98	33		2	4	73 (2)	10	11	2	22 (2)	5	4
Heart failure	47	13		1		10 (1)	7	30		2	2	9
Dyslipidemia	41	16			1	23	11	6		10	4	2
Ischemic heart disease	33	11		5		2	12 (3)	19 (2)			5 (2)	6 (2)
Atrial fibrillation	12	7		1		2	3	7 (1)		1	3	3 (1)
Stroke	5	2		1				5 (1)				2 (1)

[1]All cases with dengue as principal or secondary diagnosis.
[2]Cases with dengue as principal diagnosis and no secondary diagnoses recorded.
[3]Cases with dengue as principal or secondary diagnosis and at least one other diagnosis recorded.
[4]Cases with dengue as principal or secondary diagnosis and at least one comorbidity defined in Table S1 recorded (subset of [3] excluding dengue symptoms and other codes not categorized).

Table S3. Summary of dengue cases for Brazil (SIHSUS 2008-2015)

					All dengue cases (deaths)				Severe dengue cases (A91)(deaths)			
**Group**	**Cases**	**Severe Dengue**	**ICU Admission**	**In-hospital deaths**	**0–8**	**9–45**	**46–60**	**≥61**	**0–8**	**9–45**	**46–60**	**≥61**
**All dengue cases[1]**	532,821	27,124	4536	2698	73,839 (237)	324,240 (964)	74,460 (529)	60,282 (968)	6005 (153)	15,843 (465)	3222 (203)	2054 (231)
**Dengue only[2]**	522,111	26,398	4133	2347	72,304 (220)	318,819 (860)	72,742 (460)	58,246 (807)	5928 (142)	15,429 (422)	3084 (177)	1957 (202)
**Dengue + any diagnosis[3]**	10,710	726	403	351	1535 (17)	5421 (104)	1718 (69)	2036 (161)	77 (11)	414 (43)	138 (26)	97 (29)
**Dengue + comorbidity[4]**	4004	283	202	198	559 (9)	1604 (50)	760 (41)	1081 (98)	40 (5)	137 (21)	63 (13)	43 (14)
Infectious diseases	1294	85	66	75	274 (7)	650 (25)	181 (18)	189 (25)	17 (4)	48 (10)	13 (5)	7 (5)
Pulmonary disorders	865	75	56	67	228	350 (13)	92 (12)	195 (42)	19	34 (7)	10 (3)	12 (6)
Hypertension	595	45	10	10	2 (1)	116	190	287 (9)		14	24	7 (1)
Urinary disorders	468	8	2	2	33	257 (1)	84	94 (1)	1	7		
Diabetes	295	25	13	7	3	61 (2)	102 (3)	129 (2)	1	4 (1)	9 (2)	11
Renal disease or failure	191	23	29	17	14 (1)	80 (6)	43 (4)	54 (6)	2 (1)	13 (2)	5 (3)	3 (1)
HIV	113	13	0	1	4	70 (1)	32	7		11	2	
Heart failure	104	5	17	14		8 (1)	20 (3)	76 (10)		3		2 (1)
Ischemic heart disease	29		4	3		1	6 (1)	22 (2)				
Stroke	19	2	5	2		3 (1)	3	13 (1)		1 (1)		1
Atrial fibrillation	13						4	9				
Obesity	12	2				7	1	4		2		
Dyslipidemia	6				1	1	2	2				

[1]All cases with dengue as principal or secondary diagnosis.
[2]Cases with dengue as principal diagnosis and no secondary diagnoses recorded.
[3]Cases with dengue as principal or secondary diagnosis and at least one other diagnosis recorded.
[4]Cases with dengue as principal or secondary diagnosis and at least one comorbidity defined in Table S1 recorded (subset of [3] excluding dengue symptoms and other codes not categorized).

Table S4. Summary of dengue cases for Colombia (RIPS 2009-2017)

Group	Cases	Severe Dengue	In-hospital deaths	All dengue cases (deaths)				Severe dengue cases (A91) (deaths)			
				0–8	9–45	46–60	≥61	0–8	9–45	46–60	≥61
**All dengue cases**[1]	77,821	14,242	260	21,408 (60)	45,112 (88)	6354 (36)	4947 (76)	4341 (49)	8278 (53)	972 (22)	651 (30)
**Dengue only**[2]	69,341	12,722	153	19,005 (44)	40,824 (59)	5574 (16)	3938 (34)	3916 (35)	7429 (35)	858 (10)	519 (16)
**Dengue + any diagnosis**[3]	8480	1520	107	2403 (16)	4288 (29)	780 (20)	1009 (42)	425 (14)	849 (18)	114 (12)	132 (14)
**Dengue + comorbidity**[4]	2979	474	67	848 (5)	1121 (16)	373 (14)	637 (32)	133 (4)	208 (10)	52 (8)	81 (7)
Infectious diseases	1039	148	23	364 (3)	481 (4)	99 (6)	95 (10)	46 (3)	77 (4)	12 (3)	13 (3)
Pulmonary disorders	768	186	26	378 (3)	213 (8)	63 (6)	114 (9)	82 (2)	71 (5)	11 (4)	22 (4)
Urinary disorders	677	61	6	128	317	82 (1)	150 (5)	11	34	9 (1)	7
Hypertension	369	49	9	3	57 (1)	89 (2)	220 (6)	1	14	11	23
Diabetes	213	37	2	2	22	68 (1)	121 (1)		4	11	22
Renal disease or failure	81	13	7	3	30	11 (2)	37 (5)		4	1 (1)	8 (3)
Obesity	49	13		8	20	14	7	1	8	4	
Ischemic heart disease	37	8	4		6	10	21 (4)		1	2	5 (1)
Heart failure	33	5	4		3 (1)	6	24 (3)		1 (1)		4 (1)
HIV	32	5	3	1	26 (3)	4	1		4 (1)	1	
Dyslipidemia	14			2	1	6	5				
Atrial fibrillation	12		1		1		11 (1)				

[1]All cases with dengue as principal or secondary diagnosis. In RIPS database a distinct hospitalization episode may consist of multiple RIPS records; 91,634 raw dengue records were identified as principal or secondary. These raw records were aggregated by identifying distinct hospitalization episodes for individual patients, irrespective of whether the episode was across multiple hospitals or departments. Of the 91,634 raw records, there were 79,910 distinct patient episodes based on overlapping admission and discharge dates. Of these, 2,089 episodes were excluded from the analysis due to inconsistent reporting of key attributes (age, sex, diagnosis codes). Thus, 77,821 hospitalized dengue cases were extracted and included in analysis.
[2]Cases with dengue as principal diagnosis and no secondary diagnoses recorded.
[3]Cases with dengue as principal or secondary diagnosis and at least one other diagnosis recorded.
[4]Cases with dengue as principal or secondary diagnosis and at least one comorbidity defined in Table S1 recorded (subset of [3] excluding dengue symptoms and other codes not categorized).

Table S5. Frequency of the top (accounting for 95% of codes) ICD-10 codes included in the infectious disease A00–A99 comorbidity category by country

ICD10	Description	BRA	COL	MEX	Total	Cumulative %
A09	Other gastroenteritis and colitis of infectious and unspecified origin	388	543	628	1559	39%
A02	Other salmonella infections	49	12	385	446	51%
A92	Other mosquito-borne viral fevers	237	125	56	418	61%
A27	Leptospirosis	65	114	116	295	68%
A41	Other sepsis	95	90	66	251	75%
A04	Other bacterial intestinal infections	220	7	3	230	81%
A01	Typhoid and paratyphoid fevers	1	12	170	183	85%
A49	Bacterial infection of unspecified site	63	29	8	100	88%
A06	Amebiasis	4	24	54	82	90%
A08	Viral and other specified intestinal infections	29	20	21	70	92%
A68	Relapsing fevers	11	45	6	62	93%
A16	Respiratory tuberculosis, not confirmed bacteriologically or histologically	2	4	25	31	94%
A46	Erysipelas	15	8	8	31	95%
A77	Spotted fever [tick-borne rickettsioses]	15	4	7	26	95%
**Total reported infectious disease codes for hospitalised dengue episodes**[#]		1294	1066	1609	3969	
**Total distinct episodes**		1294	1039	1550	3883	

BRA, Brazil; COL, Colombia; MEX, Mexico.

[#]It is possible for multiple comorbidity codes to be reported in COL and MEX for a single dengue episode so the sum of the counts of infectious disease codes is higher than the number of distinct episodes (as indicated in the column totals).

Table S6. Case fatality rates and relative mortality rate in patients with hospitalized dengue and comorbidities

	Mexico				Brazil				Colombia			
**Comorbidity**	**Deaths/ Cases**	**CFR (%)**	**RMR (95% CI)**	**P**	**Deaths/ Cases**	**CFR (%)**	**RMR (95% CI)**	**P**	**Deaths/ Cases**	**CFR (%)**	**RMR (95% CI)**	**P**
**0–8 years**												
Renal disease or failure	1/23	4.3	26 (3–199)	*	1/14	7.1	23 (4–156)	*				
Pulmonary disorders	4/155	2.6	16 (5–50)	***	0/228							
Infectious diseases	7/287	2.4	15 (6–39)	***	7/274	2.6	8 (4–18)	***				
**9–45 years**												
Heart failure	1/10	10.0	98 (15–644)	*	1/8	12.5	46 (8–290)	*				
HIV	6/95	6.3	62 (27–142)	***	1/70			ns	3/26	11.5	80 (27–239)	***
Pulmonary disorders	14/260	5.4	53 (29–95)	***	13/350	3.7	14 (8–24)	***	8/213	3.8	26 (13–54)	***
Renal disease or failure	13/246	5.3	52 (28–95)	***	6/80	7.5	28 (13–60)	***				
Obesity	2/73	2.7	27 (7–109)	**	0/7							
Infectious diseases	23/988	2.3	23 (14–38)	***	25/650	3.8	14 (10–21)	***	4/481	0.8	6 (2–16)	**
Diabetes	4/466	0.9	8 (3–23)	**	2/61	2.9	12 (3–48)	*				
**46–60 years**												
Ischemic heart disease	3/12	25.0	136 (42–441)	***	1/6	16.7	26 (4–158)	*				
Pulmonary disorders	5/63	7.9	43 (15–125)	***	12/92	13.0	21 (12–35)	***	6/63	9.5	33 (13–82)	***
Heart failure	0/7				3/20	15.0	24 (8–68)	***				
Renal disease or failure	3/84	3.6	19 (5–70)	***	4/43	9.3	15 (6–38)	***	2/11	18.2	63 (16–243)	***
Infectious diseases	2/161	1.2	7 (1–31)	*	18/181	9.9	16 (10–25)	***	6/99	6.1	21 (8–53)	***
Diabetes	4/621	0.6	4 (1–11)	*	3/102	2.9	5 (2–14)	*				
Hypertension									2/89	2.2	8 (2–34)	*
**≥61 years**												
Stroke	1/5	20.0	46 (7–292)	*	1/13			ns				
Atrial fibrillation	1/7	14.3	33 (5–221)	*	0/9							
Ischemic heart disease	2/19	10.5	24 (6–102)	**	2/22	9.1	7 (2–25)	*	4/21	19.0	22 (9–57)	***
Pulmonary disorders	8/95	8.4	19 (8–48)	***	42/195	21.5	16 (12–21)	***	9/114	7.9	9 (4–19)	***

Renal disease or failure	6/87	6.9	16 (6–42)	***	6/54	11.1	8 (4–17)	***	5/37	13.5	16 (6–38)	***
Infectious diseases	4/114	3.5	8 (3–25)	**	25/189	13.2	10 (7–14)	***	10/95	10.5	12 (6–24)	***
Heart failure	0/30				10/76	13.2	9 (5–17)	***	3/24	12.5	14 (5–44)	**
Diabetes	12/412	2.9	7 (3–15)	***	2/129			ns				
Hypertension	6/376	1.6	4 (1–10)	*	9/287	3.1	2 (1–4)	*	6/220	2.7	3 (1–7)	*
Urinary disorders									5/150	3.3	4 (2–10)	*

Comorbidities only reported for age groups with at least 5 cases and 1 death, and $p<0.05$ from the Fisher’s exact test.
CFR, case fatality rate; RMR, relative mortality rate for dengue with comorbidity vs dengue alone.
Significance for the RMR of dengue with comorbidity vs dengue alone: *** $p<0.001$; ** $p<0.01$; *$p<0.05$ (Fisher’s exact test).

Figure S1. Prevalence of comorbidities in hospitalized dengue cases compared to non-dengue cases in Mexico (A), Brazil (B) and Colombia (C), grouped by age and comorbidity category. Mexico (N=15,512 dengue, N=7.35 million non-dengue), Brazil (N=10,710 dengue, N=11.8 million non-dengue), Colombia (N=8480 dengue, N=1.40 million non-dengue). Only comorbidities with relative prevalence > × 1 and p<0.01 [Fisher's exact test]) compared to non-dengue cases are shown. Relative prevalence of comorbidities in dengue cases compared the non-dengue cases is indicated above the dengue cases bar. There were no dengue comorbidities with relative prevalence > × 1 and p<0.01 compared to non-dengue cases in those aged 0–8 years in Colombia.

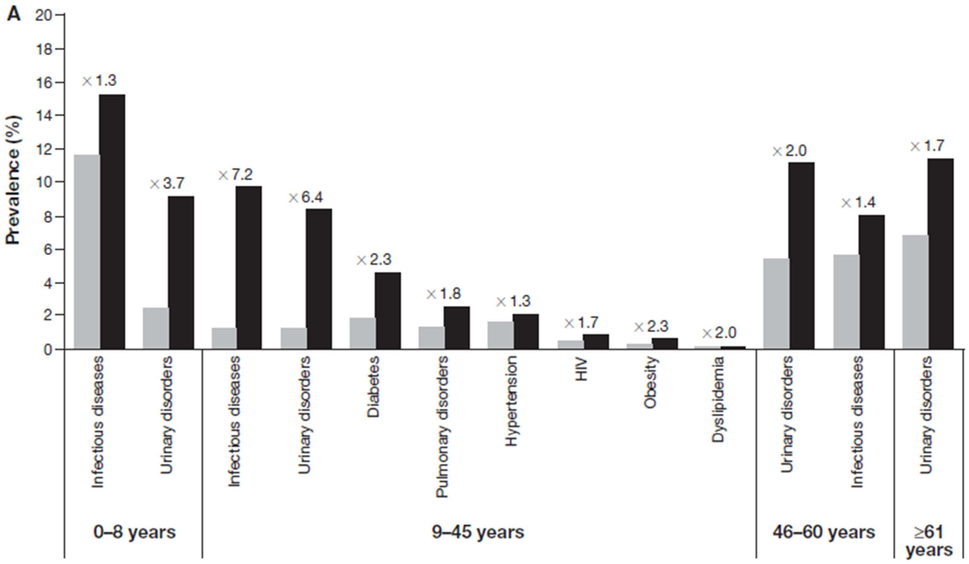

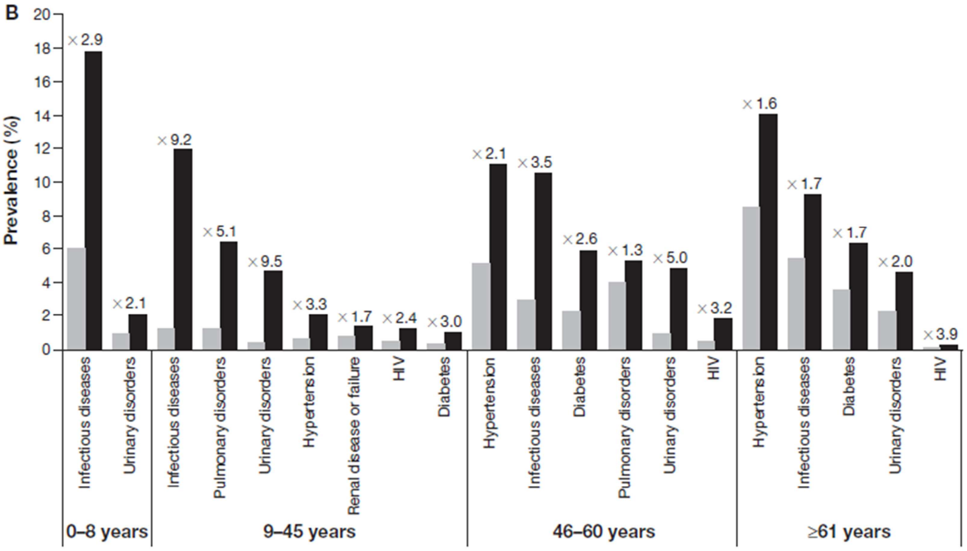
B
Prevalence (%)
20
18
16
14
12
10
8
6
4
2
0
× 2.9
× 2.1
× 9.2
× 5.1
× 9.5
× 3.3
× 1.7
× 2.4
× 3.0
× 2.1
× 3.5
× 2.6
× 1.3
× 5.0
× 3.2
× 1.6
× 1.7
× 1.7
× 2.0
× 3.9
Infectious diseases
Urinary disorders
Infectious diseases
Pulmonary disorders
Urinary disorders
Hypertension
Renal disease or failure
HIV
Diabetes
Hypertension
Infectious diseases
Diabetes
Pulmonary disorders
Urinary disorders
HIV
Hypertension
Infectious diseases
Diabetes
Urinary disorders
HIV
0–8 years
9–45 years
46–60 years
≥61 years

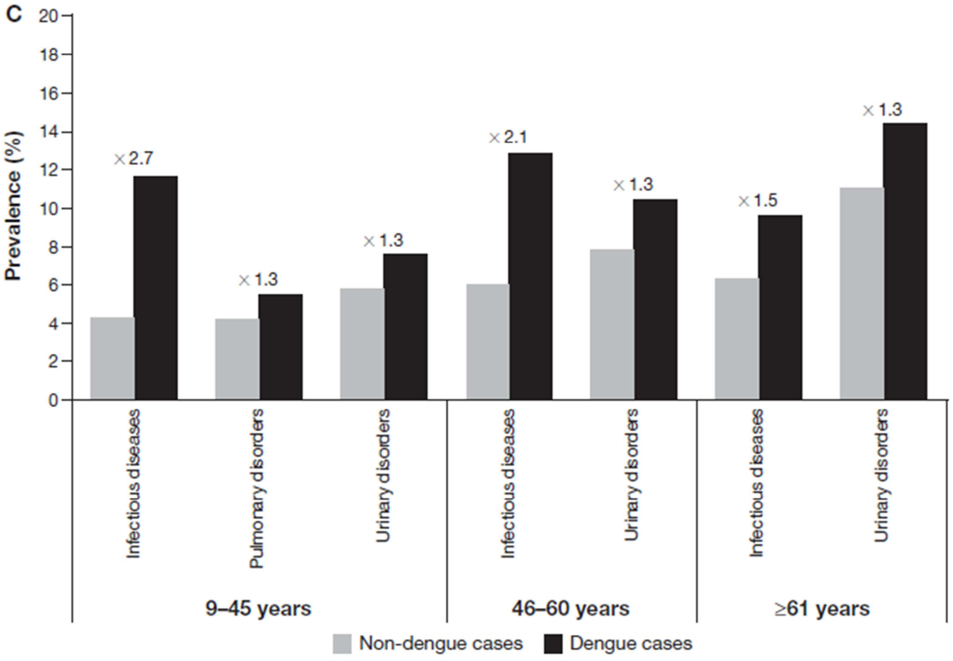
C
Prevalence (%)
20
18
16
14
12
10
8
6
4
2
0
× 2.7
× 1.3
× 1.3
× 2.1
× 1.3
× 1.5
× 1.3
Infectious diseases
Pulmonary disorders
Urinary disorders
Infectious diseases
Urinary disorders
Infectious diseases
Urinary disorders
9–45 years
46–60 years
≥61 years
Non-dengue cases
Dengue cases
